# A Comparative Study of Electronic, Optical, and Thermoelectric Properties of Zn-Doped Bulk and Monolayer SnSe Using Ab Initio Calculations

**DOI:** 10.3390/nano13142084

**Published:** 2023-07-16

**Authors:** Najwa Al Bouzieh, Muhammad Atif Sattar, Maamar Benkraouda, Noureddine Amrane

**Affiliations:** 1 Physics Department, College of Science, United Arab Emirates University (UAEU), Al Ain 15551, United Arab Emirates; 202090127@uaeu.ac.ae (N.A.B.);; 2 National Water and Energy Center (NWEC), United Arab Emirates University (UAEU), Al Ain 15551, United Arab Emirates

**Keywords:** tin selenide, DFT, electronic, structural, optical, thermoelectric, monolayer

## Abstract

In this study, we explore the effects of Zn doping on the electronic, optical, and thermoelectric properties of α-SnSe in bulk and monolayer forms, employing density functional theory calculations. By varying the doping concentrations, we aim to understand the characteristics of Zn-doped SnSe in both systems. Our analysis of the electronic band structure using (PBE), (SCAN), and (HSE06) functionals reveals that all doped systems exhibit semiconductor-like behavior, making them suitable for applications in optoelectronics and photovoltaics. Notably, the conduction bands in SnSe monolayers undergo changes depending on the Zn concentration. Furthermore, the optical analysis indicates a decrease in the dielectric constant when transitioning from bulk to monolayer forms, which is advantageous for capacitor production. Moreover, heavily doped SnSe monolayers hold promise for deep ultraviolet applications. Examining the thermoelectric transport properties, we observe that Zn doping enhances the electrical conductivity in bulk SnSe at temperatures below 500 K. However, the electronic thermal conductivity of monolayer samples is lower compared to bulk samples, and it decreases consistently with increasing Zn concentrations. Additionally, the Zn-doped 2D samples exhibit high Seebeck coefficients across most of the temperature ranges investigated.

## 1. Introduction

Due to the rapid development of applied material science, materials must be non-toxic, inexpensive, eco-friendly, efficient, and appropriate for their intended purposes. Chalcogenides have shown great potential, with SnSe being one of the most promising due to its unique electronic and thermal properties. Its band gap is suitable for solar cells with direct and indirect values of 1.30 and 0.90 eV [1], respectively. Hence, SnSe is ideal for use in photovoltaics, radiation detectors, and solid-state batteries [2,3,4,5]. Moreover, SnSe has low thermal conductivity that is attributed to the weak interlayer interactions between Sn and Se layers, resulting in a satisfactory thermoelectric performance [6].

For a material to be considered thermoelectric, it must have an excellent figure of merit (ZT) value, which is a dimensionless quantity used to quantify the performance of thermoelectric materials [7,8]. The ZT value is determined by three main characteristics of the material, the Seebeck effect (S), the electrical conductivity (σ), and the thermal conductivity ($\;k_{e}+k_{l}$), where $k_{e}$ represents the electrical thermal conductivity and $\;k_{l}$ represents the lattice thermal conductivity. To achieve high ZT value, it is necessary to decouple S, σ, and $\;k_{e}$ as these three characteristics tend to affect each other. Pristine SnSe stands out among thermoelectric materials with a high ZT value of ≅2.6 at 923 K [9], thus making SnSe desirable for research into inexpensive thermoelectric devices.

The SnSe monolayer is one of the low-dimensional materials that have shown potential to outperform bulk materials in terms of thermoelectric (TE) properties due to differences in phonon scattering mechanisms. It is gaining significant attention in the IV-VI semiconductor field because of its unique physical properties. SnSe monolayer has a graphene-like structure, with Sn and Se atoms exhibiting puckered surfaces due to sp2 hybridization [1,10], and each Sn atom forms three covalent bonds with Se, resulting in strong anisotropic properties [11,12]. In bulk α-SnSe, the atoms are arranged in an orthorhombic GeS structure with a Pnma space group at room temperature, forming double layer zigzag planes of Sn and Se atoms, which are weakly connected by van der Waals forces because of the covalent bonding within SnSe layers [13].

The electrical, thermal, and optical properties of bulk and monolayered SnSe can be altered through substitutional doping with suitable elements. For instance, a study by Zhang et al. found that Zn-doping p-type SnSe led to a ZT value of 0.96 at 873 K, which is 41% higher than that of pure SnSe [14]. Sheini also reported that Zn-doped SnSe films had higher efficiency than undoped SnSe films [15]. However, it is still unclear how Zn-doping affects the properties of SnSe monolayers. Therefore, this study was conducted to examine and compare the structural, electronic, optical, and thermoelectric properties of Zn-doped bulk SnSe (6%, 13%, 19%, and 25%) and Zn-doped monolayer SnSe (6%, 11%, 17%, and 22%) using density functional theory (DFT), with the aim of improving understanding of the properties of low-dimensional SnSe-based materials.

This study analyzes the structural characteristics of the doped bulk and monolayer SnSe compounds and compares it with the undoped SnSe crystals. The band structures and density of states were computed and compared for all systems to examine electronic properties. The thermoelectric properties of the doped structures showed a high Seebeck coefficient for both phases; however, the 3D doped systems showed an enhancement in electrical conductivity compared to the 2D systems. Optical properties were determined by studying the absorption coefficient α(ω), reflectivity R(ω), refractive index n(ω), extinction coefficient K(ω), and loss energy L(ω). Heavily Zn-doped SnSe monolayer samples were identified to have potential for UV applications. This paper highlights the possible applications of Zn-doped SnSe in both bulk and monolayer forms.

## 2. Computational Details

To investigate the impact of Zn doping on the physical properties of SnSe, first-principles calculations were carried out using the Vienna Ab-initio Simulation Package (VASP) [16]. The projector augmented plane wave method (PAW) [17] and the Perdew–Burke–Ernzerhof (PBE) generalized gradient approximation (GGA) [18] were used to calculate the exchange-correlation energy. To obtain more precise results, the strongly constrained and appropriately normed (SCAN) function was used for electronic band gap and thermoelectric calculations, as it performs well for covalent systems [19]. Additionally, the Heyd–Scuseria–Ernzerhof 06 (HSE06) screened hybrid functionals [20] were used with a 0.2 screening parameter for pure and doped systems to gain a better understanding of the obtained band gaps. A supercell of 1 × 2 × 2 was created to simulate Zn doping on bulk SnSe, while a 3 × 3 supercell was used for monolayer SnSe. The energy cutoff for the plane-wave expansion of wave functions was set at 450 eV, and the maximum force allowed for each atom was 0.02 eV/A. A vacuum spacing perpendicular to the plane was employed in SnSe monolayer systems to avoid coupling between neighboring cells. The van der Waals interaction in the supercell was accounted for using DFT-D3 [21]. A Monkhorst–Pack grid [22] of 4 × 6 × 6 was used for bulk SnSe optimization, while 4 × 1 × 4 was used for SnSe monolayer systems. For the calculation of electronic densities of states (DOS), denser K-mesh grids of 8 × 12 × 12 and 8 × 1 × 8 were used for bulk SnSe and monolayer systems, respectively. To determine the optical properties, the frequency-dependent dielectric response theory [23] was used, and VASPKIT [24] and VESTA [25] were used to analyze optical and electron redistribution, respectively. The Boltzmann transport equation was used for thermoelectric properties calculations using the BoltzTrap2 code [26], assuming a constant relaxation time. While the constant relaxation time approximation simplifies the calculations and enables efficient exploration of the thermoelectric properties, it is important to acknowledge that this assumption neglects the energy and momentum dependence of relaxation processes. Actual relaxation spectra are known to be energy and momentum dependent, resulting in mobility and conductivity dependence on an applied bias, a Fermi level position, and other factors. Thus, the constant relaxation time approximation, while computationally expedient, may not fully capture the complexity of the relaxation mechanisms in SnSe systems. To perform thermoelectric calculations on the studied monolayer systems, a more compact k grid of 20 × 1 × 20 was utilized during the non-self-consistent field calculations. This enabled the determination of Kohn–Sham energies necessary for computing the transport properties, employing the BoltzTraP2 code.

## 3. Results

### 3.1. Structural Properties

**Figure 1:**
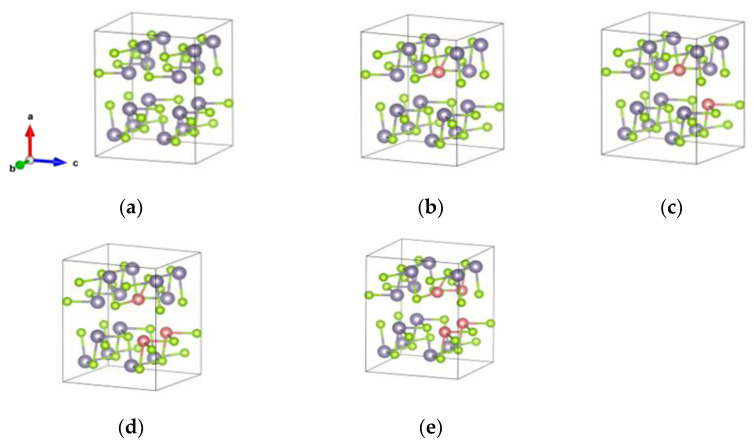
Structures of bulk *SnSe* compounds: (**a**) pure SnSe, (**b**) $Zn_{0.06}\;Sn_{0.94}Se$, (**c**) $Zn_{0.13}\;Sn_{0.87}Se$, (**d**) $Zn_{0.19}\;Sn_{0.81}Se$, and (**e**) $Zn_{0.25}\;Sn_{0.75}S.\;$ The gray balls denote Sn atoms, the green balls denote Se atoms, and the pink balls denote Zn atoms.

**Figure 2:**
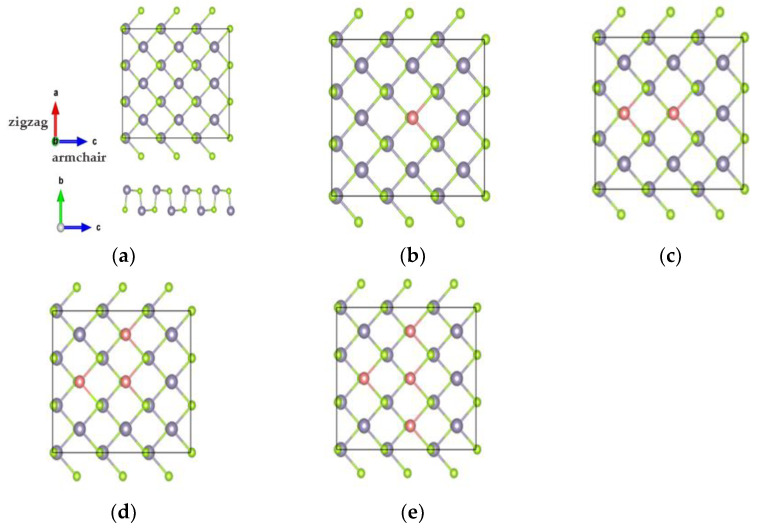
Top and side view of 3×3  *SnSe* monolayer compounds: (**a**) pure SnSe, (**b**) $Zn_{0.06}\;Sn_{0.94}Se$, (**c**) $Zn_{0.11}\;Sn_{0.89}Se$, (**d**) $Zn_{0.17}\;Sn_{0.83}Se$, and (**e**) $Zn_{0.22}\;Sn_{0.78}Se$. Sn atoms are represented by grey balls, Se atoms by green balls, and Zn atoms by pink balls.

Figure 1 shows the crystal structures of bulk SnSe systems. The lattice parameters were determined to be a = 11.78 $A^{{}^{\circ}}$, b = 4.22 $A^{{}^{\circ}}$, and c = 4.52$\;A^{{}^{\circ}}$ by relaxing the bulk SnSe unit cell; these obtained values agree with earlier theoretical calculations [27] and experimental values [28]. Figure 2 shows a puckered black phosphorus-like surface of SnSe monolayer systems, which have three covalent bonds between Sn and Se atoms. The lattice parameters in the orthorhombic monolayer were reported to be a = 4.30 $A^{{}^{\circ}}\;$ and b = 4.36 $A^{{}^{\circ}}$, which are in agreement with previous calculations [29,30]. The relaxed lattice parameters for the bulk crystals are provided in Table 1, while the relaxed lattice parameters for the monolayer structures are provided in Table 2. Tables S1 and S2 provide all bond angles for all systems (Supplementary Materials).

According to Table 1 and Table 2, the increase in Zn concentration led to an increase in the lattice parameter c for bulk and monolayer systems. Conversely, the lattice parameter b in the bulk systems and a in the monolayer systems decreased as the Zn content increased. For bulk compounds, the lattice parameter a remained mostly constant, but it decreased to 11.70 Å when the Zn concentration was 25%. This change in lattice parameter size could be attributed to the smaller Zn ion radius (0.88 Å) compared to the Sn ion radius (0.93 Å).

We have also assessed the formation energies for all the 3D and 2D Zn-doped SnSe structures considered in our study to evaluate the feasibility of achieving these doping concentrations experimentally. The formation energies were found to be negative across all concentrations, indicating that these doped structures are energetically favorable. A detailed plot of the calculated formation energies can be found in the Supplementary Materials (Figure S1).

### 3.2. Electronic Properties

**Figure 3:**
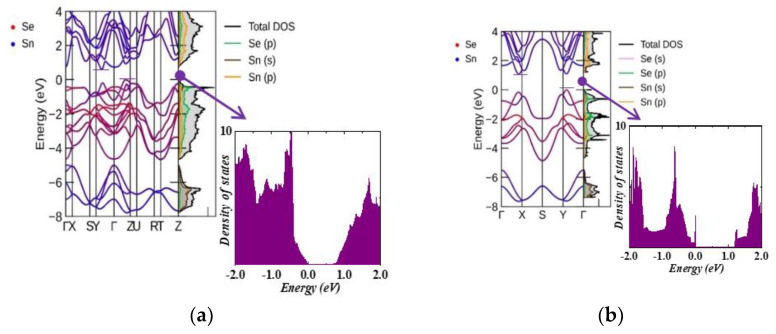

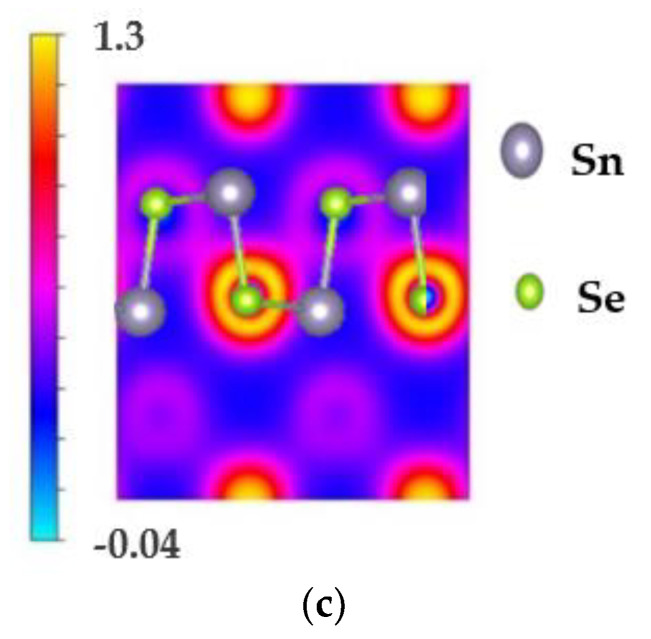
Band structure and partial DOS for (**a**) bulk α−SnSe, (**b**) monolayer α−SnSe, and (**c**) 2D charge density for SnSe with high and low charge densities, which are shown in yellow and cyan colors, respectively.

Figure 3 shows a comparison of the band structure and projected DOS (PDOS) of the unit cell of bulk SnSe and its monolayer. Sn p-states contribute the most among the conduction bands, while Se p-states dominate in undoped systems in the valence bands. Using the HSE06 functional, both structures were reported to have an indirect band gap of 1.078 eV for bulk SnSe and 1.471 eV for monolayer SnSe, which are consistent with previous studies [31,32,33]. The SnSe monolayer has a larger band gap than the bulk SnSe because of the weak interaction between the layers owing to bipolar conduction that can be effectively suppressed at high temperatures, thus resulting in improved thermoelectric properties [34]. The electronic band gap was calculated for all structures using SCAN functionals and GGA/PBE potentials, as listed in Table 3 and Table 4. The conduction band minimum (CBM) of bulk SnSe was found in the Y-Γ region, and the valence band maximum (VBM) was located in the Γ-Z region. For the monolayer SnSe, CBM was reported on the Γ-X path and the VBM in the Y-Γ direction.

Figure 3c shows the charge density of SnSe, where the electron density is higher around Se atoms than around Sn atoms, which indicates electron movement from Sn atoms to Se atoms. The Sn–Se bond is possibly a polar covalent bond with strong polarity because of the significant electronegativity difference between the Se and Sn atoms. Furthermore, the electron densities of the non-bonding neighbors Sn and Se are high. This directs that the nonbonding interactions are stronger than the typical van der Waals interaction, although they are weaker than the bond between Sn and Se atoms.

**Figure 4:**
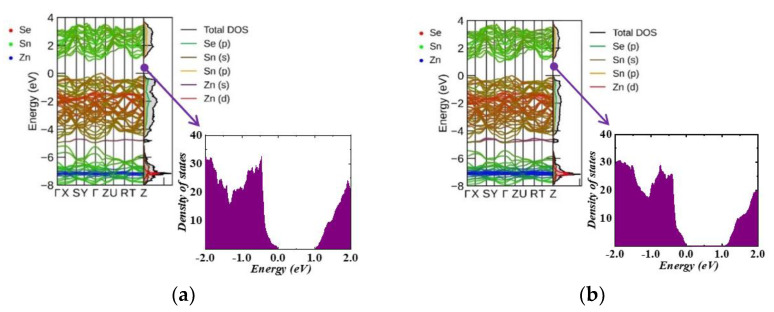

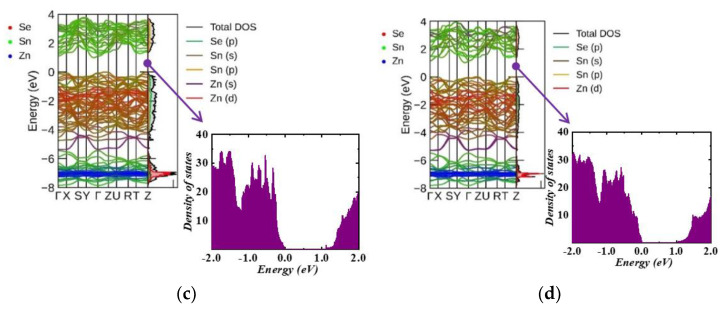
Band structure and partial DOS for Zn-doped bulk systems: (**a**) $Zn_{0.06}\;Sn_{0.94}Se$, (**b**) $Zn_{0.13}\;Sn_{0.87}Se,$ (**c**) $Zn_{0.19}\;Sn_{0.81}Se$, and (**d**) $Zn_{0.25}\;Sn_{0.75}Se$.

**Figure 5:**
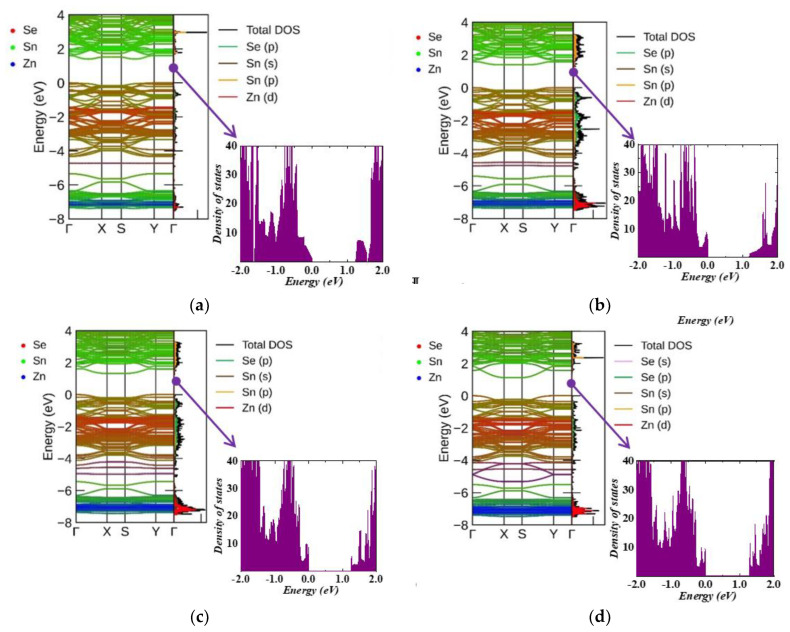
Band structure and partial DOS for Zn-doped monolayer systems: (**a**) $Zn_{0.06}\;Sn_{0.94}Se$, (**b**) $Zn_{0.11}\;Sn_{0.89}Se,$ (**c**) $Zn_{0.17}\;Sn_{0.83}Se$, and (**d**) $Zn_{0.22}Sn_{78}Se$.

Figure 4 (bulk compounds) and Figure 5 (monolayer compounds) show the band structure and PDOS of Zn-doped structures. Similar to the undoped SnSe systems, the 3D doped systems exhibit VBM in the Γ-Z region and CBM in the Y–Γ direction. However, in 2D systems, the VBM was reported at the Y point for all doped structures. The CBM was found in the Γ-X region for 6% and 11% Zn-doped SnSe, and at the X point for Zn concentrations of 17% and 22%. Figure 4 and Figure 5 show that the Zn (3d) state exhibits a strong hybridization with the Sn (5s) state at around −7 eV, while the Zn (4s) state contributes to the valence band energy at around −4 eV. As shown in Figure 5, in monolayer systems, Zn doping causes the CBM to become flatter with an increase in Zn concentration, resulting in a decreased band gap value as shown in Table 4.

**Figure 6:**
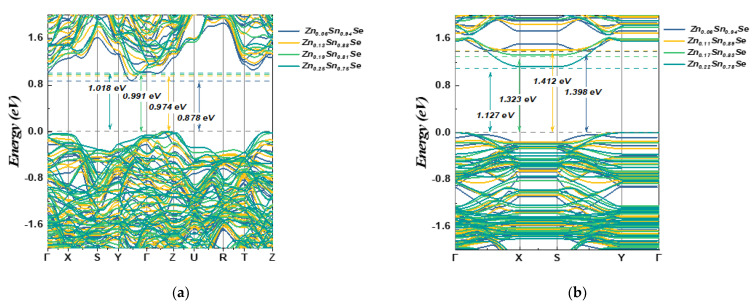
Comparative band-structure between (**a**) Zn-doped bulk SnSe compounds and (**b**) Zn-doped monolayer SnSe compounds using SCAN functional.

Figure 6 shows a comparative illustration for band structure topology that shows the band gap values for all structures. All doped and undoped systems exhibit semiconductor behavior. However, an interesting observation is that the band gap in monolayer systems decreases with the increase in the Zn concentration, while the opposite trend is observed in 3D systems. This contrasting behavior can be attributed to the inherent differences between the 2D and 3D systems and the nature of interaction with the Zn dopants. In the 2D monolayer system, Zn atoms, primarily located in the surface, significantly influence the electronic states leading to a decrease in the band gap. Moreover, an interesting evolution of the conduction band minimum (CBM) occurs with an increasing Zn concentration. For lower concentrations (Zn 6% and 11%), the CBM becomes flatter, and the band gap increases, indicative of an increase in the effective mass of the charge carriers (Tables S3 and S4 in Supplementary Materials). However, upon further increasing the Zn concentration (Zn 17% and 22%), we observe a shift in this trend. The initially flattened CBM evolves to present a higher curvature, suggesting a decrease in the effective mass of the charge carriers. This change indicates a transition in the electronic band structure, with the merging of two peaks into a single band at higher Zn concentration. The evolution of the CBM and its associated effective mass directly impacts the mobility of the charge carriers. A flatter band (higher effective mass) could potentially result in reduced carrier mobility, potentially leading to decreased electrical conductivity. In contrast, an increase in curvature (decreased effective mass) would enhance carrier mobility, thereby positively affecting electrical conductivity, subject to factors, such as overall carrier concentration. In terms of thermoelectric properties, the effective mass plays a crucial role. An increased effective mass can enhance the Seebeck coefficient, potentially improving the thermoelectric performance of the material.

In the 3D bulk system where spatial confinement is less profound, the Zn dopants exert a more diffused influence, affecting the band edges less directly. This diffused influence, potentially coupled with lattice strain or deformation due to Zn introduction, may result in an increase in the band gap. The dimensionality of the system, dopant interaction, changes in carrier effective mass, and subsequent alterations in carrier mobility and thermoelectric properties together contribute to the observed difference in the bandgap behavior upon Zn doping in the monolayer and bulk SnSe.

**Figure 7:**
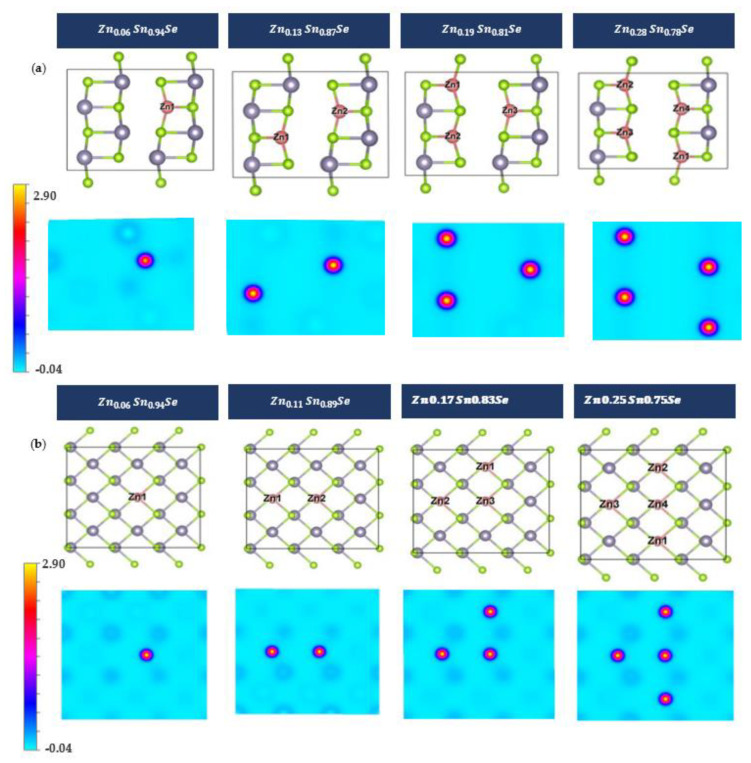
Charge density for (**a**) 3D SnSe compounds and (**b**) 2D SnSe compounds, with high and low charge densities shown in yellow and cyan colors, respectively. (**a**,**b**) $Zn_{0.13}\;Sn_{0.87}Se$.

As shown in Figure 7, the charge density illustrations show that the projected charge density of 2D Zn-doped systems demonstrates an enhanced bonding between Zn and Se compared to 3D systems.

### 3.3. Optical Properties

The optical properties, which determine the propagation of electromagnetic radiation in the material and its linear response to it [36], can be examined by studying the material’s dielectric function, which is defined as follows [37]:

(1) $$\varepsilon \left(\omega \right)=\varepsilon_{1}\left(\omega \right)+i\varepsilon_{2}\left(\omega \right)={\left(n+iK\left(\omega \right)\right)}^{2}$$

**Figure 8:**
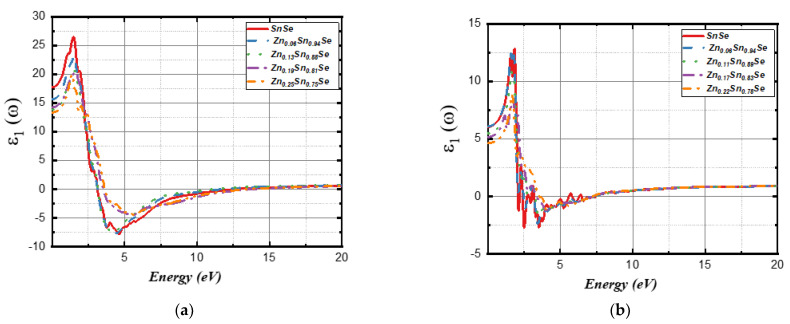

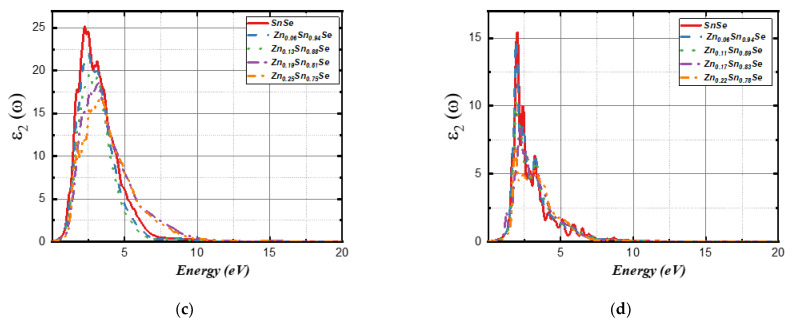
The measured real part $\varepsilon_{1}\left(\omega \right)$ of the dielectric constant (**a**,**b**) and the imaginary part $\varepsilon_{2}\left(\omega \right)$ of the dielectric constant (**c**,**d**) for 3D SnSe compounds (left side) and 2D SnSe compounds (right side).

This function has two parts: the real part $\varepsilon_{1}\left(\omega \right)$ and the imaginary part $\varepsilon_{2}\left(\omega \right)\;$ of the dielectric constant. Figure 8 shows dielectric function parts for 3D and 2D compounds based on incident photon energy. The curves of $\varepsilon_{1}\left(\omega \right)$ and $\varepsilon_{2}\left(\omega \right)\;$ for all systems are highly anisotropic in the directions a, b, and c (see Supplementary Materials, Figures S2–S4). The 2D systems have a smaller dielectric constant than 3D systems, and bulk materials show optical transitions compared to 2D structures due to interlayer distances [38]. The static dielectric constant, represented by $\varepsilon_{1}\left(0\right),\;$ for undoped 3D SnSe, as demonstrated in Figure 8a, was measured to be 17.72. This dielectric constant is dependent on the refractive index at frequencies above the lattice vibration frequency. It dropped to 15.64, 14.22, 13.84, and 13.35 when the Zn concentration was increased to 6%, 13%, 19%, and 25%, respectively. $\varepsilon_{1}\left(0\right)$ of the monolayer systems was 6.08 for the undoped system when the Zn concentration was 6%; it changes to 5.50, 5.15, and 4.62 when Zn concentrations increase to 11%, 17%, and 22%, respectively. All SnSe structures could be used for capacitors applications, as all the studied structures have high static dielectric constants.

**Figure 9:**
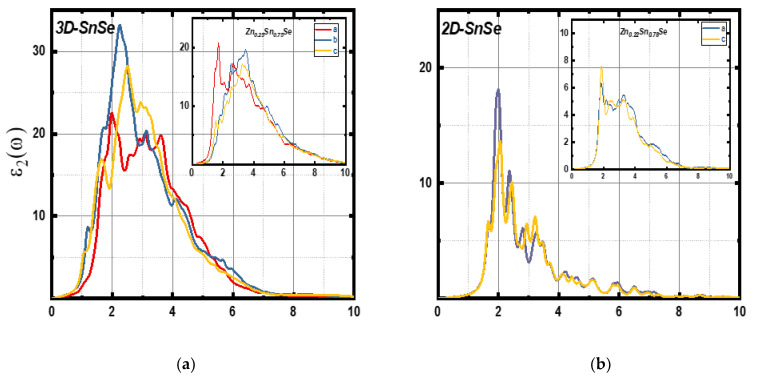
Calculated imaginary part $\varepsilon_{2}\left(\omega \right)$ of the dielectric function for (**a**) 3D undoped SnSe and Zn (25%)-doped 3D SnSe and (**b**) 2D undoped SnSe and Zn (22%)-doped 2D SnSe along a, b, and c directions.

Figure 8c,d represents the imaginary part $\varepsilon_{2}\left(\omega \right)$ for bulk and monolayer systems. As shown in the spectra, 3D SnSe displays two peaks in the visible region, one at 2.23 eV and the other at 3.13 eV. The Zn-doped 3D structure (6% and 13%) exhibited one peak at ~2.60 eV and another at ~3.10 eV. In comparison, the Zn-doped 3D structure (19% and 25%) exhibited one peak at ~3.30 eV and another at ~2.55 eV. All these peaks were observed in the visible light region. Moreover, all bulk systems exhibit high $\varepsilon_{2}\left(\omega \right)$ peaks in the (zigzag) b direction than the (armchair) c and a direction, except for Zn (25%), as the peak in the a direction, which was located at 1.70 eV, was higher than the b and c paths, as shown in Figure 9a.

Figure 8d shows $\varepsilon_{2}\left(\omega \right)$ for 2D SnSe systems. As shown in the figure, the undoped monolayer has three peaks that are located at 2.02 eV, 2.39 eV, and 3.23eV, while the doped monolayer systems have little red-shifted peaks as the Zn concentration increases. Moreover, undoped SnSe, Zn (6%), and Zn (11%) show higher $\varepsilon_{2}\left(\omega \right)\;$ peaks in the a (zigzag) direction compared to the c (armchair) direction. However, the peak on the c direction becomes higher and is located at 1.87 eV as the Zn concentration increases, as shown in Figure 9b. According to $\varepsilon_{2}\left(\omega \right)$ curves, all the studied SnSe structures demonstrate semiconducting properties, along with the DOS and band structure plots in Figure 3 and Figure 4.

The optical properties of materials can be better understood by reviewing absorption coefficient $\alpha \left(\omega \right)$, refractive index $n\left(\omega \right)$, reflectivity $R\left(\omega \right)$, extinction $K\left(\omega \right)$, and the loss energy function $L\left(\omega \right)\;$ [39]:

(2) $$\alpha \left(\omega \right)=\frac{\sqrt{2}\omega }{c}\;{\left(\sqrt{ [\varepsilon_{1}^{2}\left(\omega \right)+\varepsilon_{2}^{2}\left(\omega \right)]}-\varepsilon_{1}\left(\omega \right)\right)}^{\frac{1}{2}}\;$$

(3) $$n\left(\omega \right)={\left(\;\frac{\sqrt{ [\varepsilon_{1}^{2}\left(\omega \right)+\varepsilon_{2}^{2}\left(\omega \right)]}+\varepsilon_{1}\left(\omega \right)}{2}\;\right)}^{\frac{1}{2}}\;$$

(4) $$R\left(\omega \right)=\frac{{\left(n\left(\omega \right)-1\right)}^{2}+K^{2}\left(\omega \right)}{{\left(n\left(\omega \right)+1\right)}^{2}+K^{2}\left(\omega \right)}\;$$

(5) $$K\left(\omega \right)={\left(\;\frac{\sqrt{ [\varepsilon_{1}^{2}\left(\omega \right)+\varepsilon_{2}^{2}\left(\omega \right)]}-\varepsilon_{1}\left(\omega \right)}{2}\;\right)}^{\frac{1}{2}}\;$$

(6) $$L\left(\omega \right)=\frac{\varepsilon_{2}\left(\omega \right)}{\varepsilon_{1}^{2}\left(\omega \right)+\varepsilon_{2}^{2}\left(\omega \right)}\;$$

**Figure 10:**
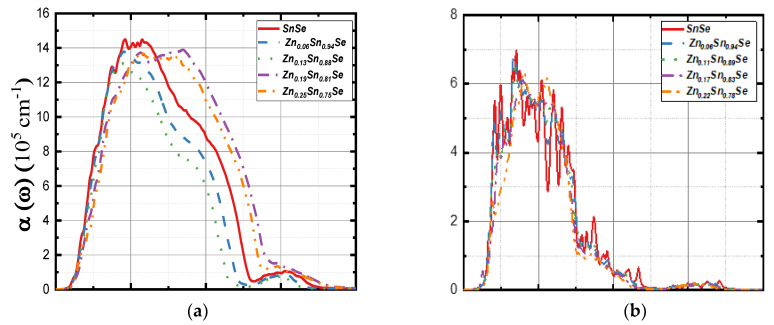

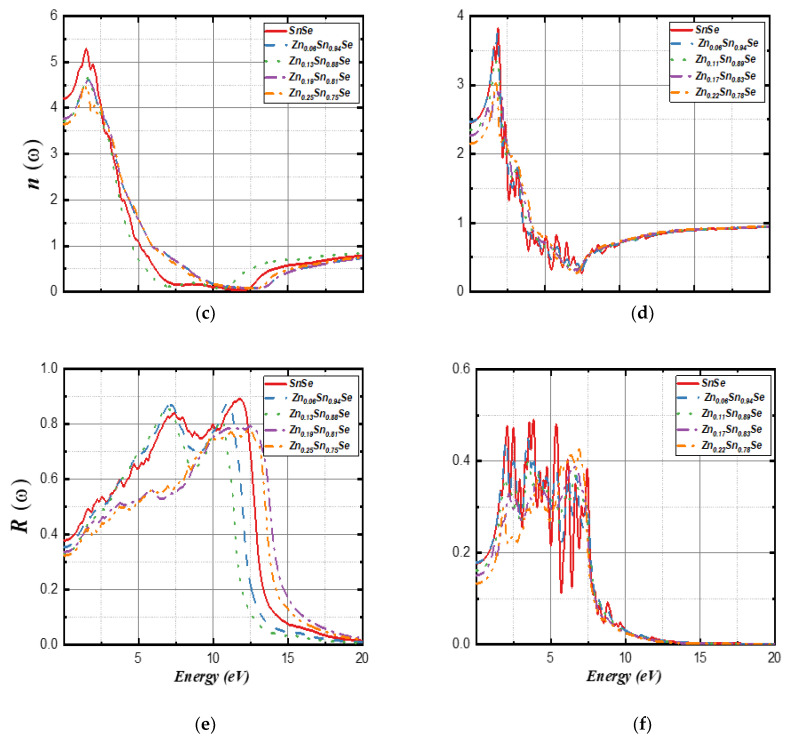
Calculated absorption coefficient $\alpha \left(\omega \right)$ (**a**,**b**), the refractive index $n\left(\omega \right)$ (**c**,**d**), and the reflectivity $R\left(\omega \right)$ (**e**,**f**) for 3D SnSe compounds (left side) and 2D SnSe compounds (right side).

Figure 10a,b shows the calculated absorption coefficients for the bulk and monolayer SnSe compounds, respectively. The spectra of bulk Zn (6%) structure exhibited a similar peak as the undoped one located at ~5.0 eV, while it becomes a broader peak at ~8 eV for Zn (19% and 25%). The monolayer compounds of SnSe demonstrated a similar zigzag pattern, where the highest peak was observed at ~3.50 eV for the undoped structure and for Zn (6% and 11%), although it shifted to ~4.0 eV for Zn (17% and 22%). The refractive index $n\left(\omega \right)$ spectra of the bulk and monolayer systems are plotted in Figure 10c,d, respectively. The value of n(0) was found around 4.21 for undoped 3D SnSe and around 3.70 for doped systems. The value of $n\left(0\right)$ for the undoped 2D SnSe monolayer was 2.47 and drops to 2.15 with the increase in Zn concentration. All these values of $n\left(0\right)$ were observed in the visible light region. Reflectivity *R(ω)* of the studied systems is shown in Figure 10e for 3D compounds and in Figure 10f for 2D compounds. For the bulk SnSe systems, the highest value of *R*(0)~0.38 was observed for an undoped structure, and the lowest value of ~0.32 was observed for Zn (25%). The zigzag trend for the reflectivity is similar for the pristine and for Zn (6% and 13%), while the peaks become wider and shifted toward the vacuum ultraviolet region of ~12 eV for Zn (19% and 25%). In Figure 10f, the undoped SnSe monolayer has several reflection peaks, which are probably created by the hybridization of Sn and Se p-states. With the increase in Zn concentration, the peaks are blue-shifted to ~7 eV for Zn (17% and 22%). This result indicates that Zn doping could have deep UV applications.

**Figure 11:**
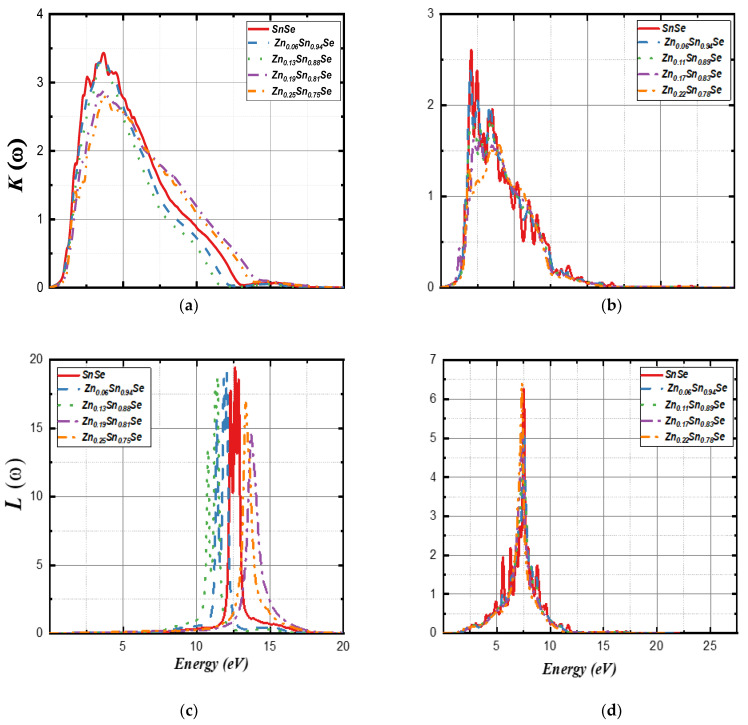
Calculated extinction coefficient $K\left(\omega \right)$ (**a**,**b**) and the energy loss $L\left(\omega \right)$ (**c**,**d**) for 3D SnSe compounds (left side) and 2D SnSe compounds (right side).

Figure 11 shows the extinction coefficient *K*(*ω*) and loss energy *L*(*ω*). The extinction coefficient trends are almost the same for doped and undoped systems; however, the peaks among the 2D systems were sharper. All $K\left(\omega \right)$ peaks for 3D compounds were at ~3.70 eV, while the undoped 2D system has three peaks at 2.01, 2.48, and 3.52 eV. Zn (6%, 11%, and 17%) also has three peaks that were located at ~2.02, 2.54, and 3.35 eV, respectively, while they were shifted to 1.88, 3.35, and 4.00 eV for Zn (22%). Figure 11c presents the loss energy for the 3D systems. The energy loss primarily occurs in the ultra-violet region as undoped SnSe has three peaks at 12.30, 12.63, and 12.85 eV. Moreover, Zn (6%) peaks were found at 11.35, 11.84, and 12.06 eV. In comparison, Zn (13%) peaks were observed at 10.77, 11.30, and 11.41 eV. For Zn (19% and 25%), the peaks were blue-shifted to 13.36 and 13.68 eV, respectively. For 2D compounds, Zn doping did not have much effect on the peaks of loss energy, as shown in Figure 11d. *L*(*ω*) peaks for all 2D systems were reported at ~7.50 eV.

### 3.4. Thermoelectric Properties

**Figure 12:**
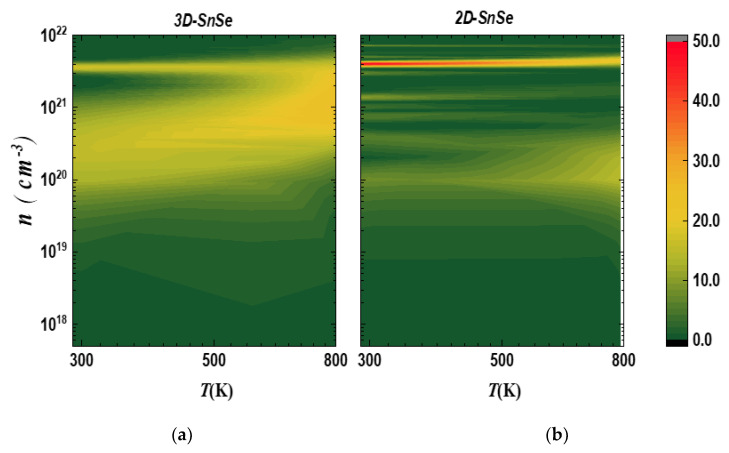
Mapping of the power factor (PF) (μV/Kcm) against carrier concentration *n* and temperature *T* for (**a**) bulk α−SnSe and (**b**) monolayer α−SnSe.

Figure 12 shows the analysis of the power factor $\left(S^{2}\sigma \right)$ for 2D and 3D SnSe systems without doping over a temperature range of 300–800 K. It was observed that for the bulk SnSe, the high values of the power factor shifted to higher carrier concentrations (*n*) across all temperature ranges. However, for the monolayer system, the power factor was found to be higher in the high *n* region at lower temperatures. In the mid-range of *n*, the higher values of the power factor were observed to shift to higher temperatures, which is consistent with previous studies [30].

**Figure 13:**
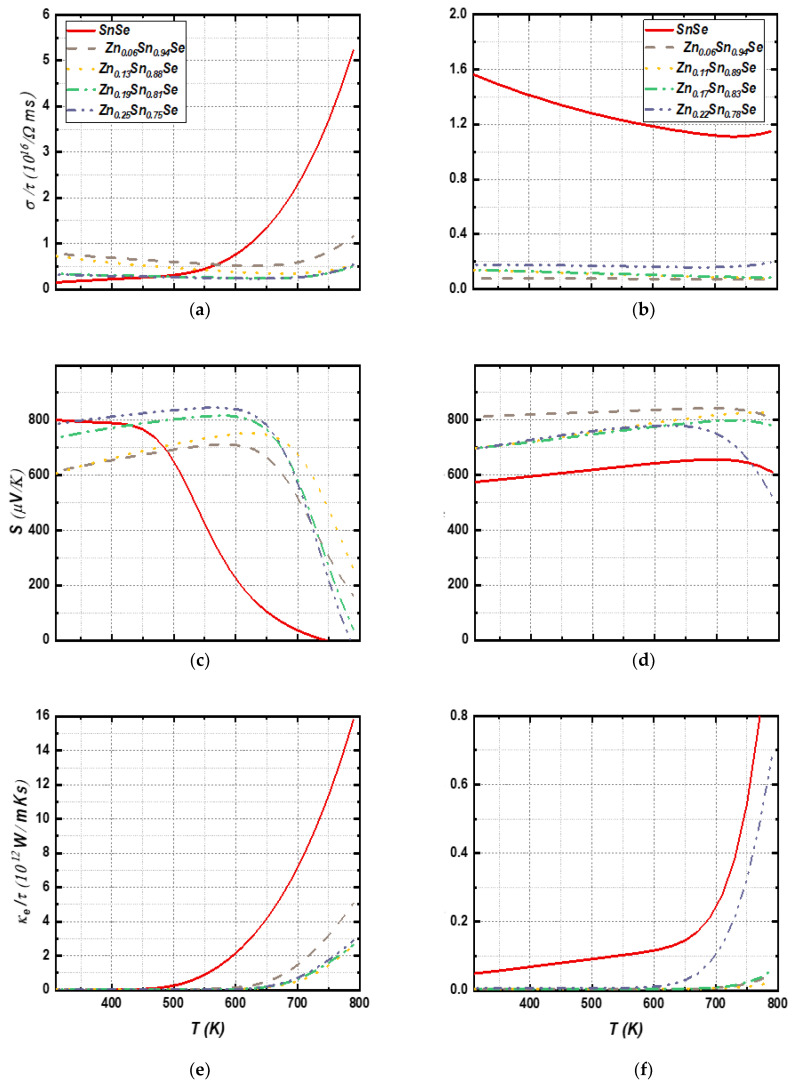
Calculated electrical conductivity (**a**,**b**), the Seebeck coefficient (**c**,**d**), and the electronic thermal conductivity (**e**,**f**) for 3D SnSe compounds (left side) and 2D SnSe compounds (right side).

The values of electrical conductivity (*σ*), Seebeck coefficient (S), and electronic thermal conductivity ($\kappa_{e}$) for both bulk and monolayer SnSe compounds at a carrier concentration $n=10^{17}{\;\mathrm{cm}}^{-3}$ were calculated, and the results are shown in Figure 13. These values can be used to assess the thermoelectric properties of Zn-doped SnSe structures. The results show that all doped bulk samples have higher electrical conductivity values than the undoped sample at temperatures below 500 K, as can be seen in Figure 13a, with Zn (6%) having the highest values of σ, which decreases with increasing Zn concentration. This trend may be related to the increased band gap observed in doped bulk structures, leading to reduced carrier mobility. However, in monolayer systems (Figure 13b), Zn doping had the opposite effect, reducing electrical conductivity. The observed reduction in electrical conductivity aligns with the observed flattening and eventual increase in the curvature of the CBM with increased Zn concentration, suggesting a decrease in the effective mass of the charge carriers and thus reduced carrier mobility. Despite the Zn doping, the electrical conductivity remained relatively constant across all temperature ranges. Furthermore, the undoped SnSe monolayer exhibited higher σ values than the undoped bulk SnSe sample in the low- and mid-range temperature regions. This contrast highlights the significant influence of dimensionality and Zn concentration on the electronic structure and subsequent transport properties. The effects of Zn doping and dimensionality on the band structure, particularly the CBM flattening and the ensuing changes in effective mass, play a critical role in the observed transport characteristics. In addition, electrical conductivity plots show an increasing trend with temperature for the bulk compounds, which is likely due to thermal excitation of carriers and additional energy levels introduced by Zn doping. Our findings align with recent studies of doped SnSe exhibiting similar temperature-dependent behavior [5].

Figure 13c,d shows the Seebeck coefficient for the bulk and monolayer systems, respectively. At temperatures below 350 K, the undoped SnSe sample had a higher S value than the doped samples, but at higher temperatures, the doped samples had higher values than the undoped one. Additionally, in the bulk systems, increasing Zn concentration led to increased S values at temperatures below 650 K, with Zn (13%) exhibiting the highest S values among the doped samples. In monolayer systems, Zn doping resulted in increased Seebeck coefficient values across most temperature ranges, with Zn (6%) having the highest S values in low- and mid-range temperatures. However, as the Zn concentration increases, the S values decrease, especially at higher temperatures. The S values increase with temperature, indicating an increase in carrier scattering near the Fermi level, which improves the semiconductor functionality.

Figure 13e,f illustrates the electronic thermal conductivity results for both bulk and monolayer SnSe compounds with Zn doping. The doped samples showed lower $\kappa_{e}$ values compared to the undoped samples. Monolayer systems exhibited lower $\kappa_{e}$ values than bulk systems. Zn (13%) exhibited the lowest $\kappa_{e}$ values among bulk structures, while Zn (11%) had the lowest values among monolayer compounds. The trend of increasing $\kappa_{e}\;$ values can be explained by the Wiedemann–Franz law $\kappa_{e}=L\sigma T$, where L represents the Lorentz constant and T represents the temperature.

**Figure 14:**
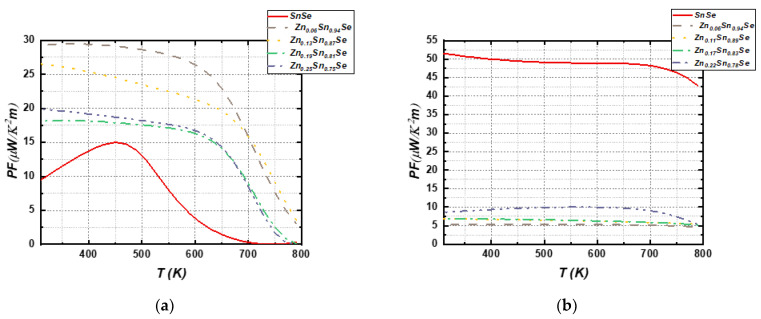
Calculated power factor (PF) for (**a**) 3D SnSe compounds and (**b**) 2D SnSe compounds (right side).

The power factor (PF) values are depicted in Figure 14a for bulk SnSe materials and in Figure 14b for monolayer counterparts. We have used constant relaxation time approximation with t = 10–14 s. In the case of three-dimensional (3D) SnSe systems, it is observed that doped configurations exhibit higher PF values across all temperature ranges, with Zn doping at a concentration of 6% showing the highest PF values among all 3D doped systems. By contrast, the power factor of undoped two-dimensional (2D) SnSe is higher than that of the doped counterparts. Furthermore, the 2D undoped SnSe sample demonstrates higher PF values compared to the 3D structures, attributed to its higher electrical conductivity characteristics.

## 4. Conclusions

This study presents a comparative analysis of the effects of Zn doping on the properties of monolayer and bulk SnSe structures. The electronic properties of the doped samples indicate that the Zn (3d) state has strong hybridization with the Sn (5s) state around −7 eV, while the Zn (4s) state contributes to the valence band energy around −4 eV. In monolayer systems, Zn doping causes the conduction band minimum (CBM) to become flatter with an increase in Zn concentration, resulting in a decreased band gap value for Zn (25%) compared to bulk doped structures. The optical properties of all doped systems demonstrate semiconducting behavior suitable for optoelectronic and photovoltaic applications. Furthermore, 2D samples exhibit smaller dielectric constants than 3D samples, making Zn-doped SnSe a viable material for capacitor fabrication. Additionally, heavily doped SnSe monolayer samples have the potential for use in deep UV applications, as shown by the reflectivity spectra.

Regarding thermoelectric transport calculations, this study demonstrates that Zn doping enhances electrical conductivity for bulk SnSe at temperatures below 500 K, while it decreases electrical conductivity in monolayer systems. However, Zn-doped 2D samples have higher Seebeck coefficient values in most temperature ranges. Additionally, all Zn-doped compounds, both bulk and monolayer, exhibit decreased electronic thermal conductivity compared to undoped samples. Overall, this work aims to provide a better understanding of the low-dimensional properties of SnSe structures, which may serve as a foundation for future applications.

## Figures and Tables

**Table 1:** DFT-calculated lattice parameters of bulk α-SnSe compounds.

Structure	$a\left(A^{{}^{\circ}}\right)$	$b\left(A^{{}^{\circ}}\right)$	$c\left(A^{{}^{\circ}}\right)$
α−SnSe	11.78	4.22	4.52
Other DFT calculations ^1^	11.72	4.20	4.55
$Zn_{0.06}\;Sn_{0.94}Se$	11.79	4.20	4.57
$Zn_{0.13}\;Sn_{0.87}Se$	11.79	4.17	4.62
$Zn_{0.19}\;Sn_{0.81}Se$	11.78	4.14	4.69
$Zn_{0.25}\;Sn_{0.75}Se$	11.70	4.10	4.82

^1^ Ref. [27].

**Table 2:** DFT-calculated lattice parameters of monolayer α-SnSe compounds.

Structure	$a\left(A^{{}^{\circ}}\right)$	$c\left(A^{{}^{\circ}}\right)$
α−SnSe	4.30	4.36
Other DFT calculations ^1^	4.29	4.40
$Zn_{0.06}\;Sn_{0.94}Se$	4.27	4.43
$Zn_{0.11}\;Sn_{0.89}Se$	4.24	4.48
$Zn_{0.17}\;Sn_{0.83}Se$	4.21	4.52
$Zn_{0.22}\;Sn_{0.78}Se$	4.14	4.58

^1^ Ref. [29].

**Table 3:** DFT-calculated band gap (eV) of bulk α-SnSe compounds.

Structure	GGA	SCAN	HSE06	Other DFT Calculations	Exp
α−SnSe	0.601	0.780	1.078	1.1 ^1^ (HSE06)	0.86 ^2^
$Zn_{0.06}\;Sn_{0.94}Se$	0.679	0.878	1.161		
$Zn_{0.13}\;Sn_{0.87}Se$	0.779	0.974	1.263		
$Zn_{0.19}\;Sn_{0.81}Se$	0.803	0.991	1.278		
$Zn_{0.25}\;Sn_{0.75}Se$	0.822	1.018	1.315		

^1^ Ref. [35]; ^2^ Ref. [33].

**Table 4:** DFT-calculated band gap (eV) of monolayer α-SnSe compounds.

Structure	GGA	SCAN	HSE06	Other DFT Calculations
α−SnSe	0.986	1.067	1.471	1.67 ^1^ (HSE06)
$Zn_{0.06}\;Sn_{0.94}Se$	1.213	1.398	1.558	-
$Zn_{0.11}\;Sn_{0.89}Se$	1.217	1.412	1.720	-
$Zn_{0.17}\;Sn_{0.83}Se$	1.120	1.323	1.600	-
$Zn_{0.22}\;Sn_{0.78}Se$	0.910	1.127	1.358	0.97 ^2^ (GGA)

^1^ Ref. [13]; ^2^ Ref. [1].

## Data Availability

Data will be made available on request.

## Supplementary Materials

The following supporting information can be downloaded at: https://www.mdpi.com/article/10.3390/nano13142084/s1, Table S1. DFT-calculated bond angles of bulk α-SnSe compounds. Table S2. DFT-calculated bond angles of monolayer α-SnSe compounds. Table S3: Effective Masses (m*) of Charge Carriers at Valence Band Maximum (VBM) and the Conduction Band Minimum (CBM) for the 2D SnSe systems. Table S4: Effective Masses (m*) of Charge Carriers at Valence Band Maximum (VBM) and the Conduction Band Minimum (CBM) for the 3D SnSe systems. Figure S1: Variation of the formation energy of doped SnSe structures with respect to the number of Zn dopant atoms. Figure S2: The measured real part ε_1_ (ω) of the dielectric constant (a and b) and the imaginary part ε_2_ (ω) of the dielectric constant (c and d) for 3D SnSe (left side) and 2D SnSe (right side). Figure S3: The measured real part ε_1_ (ω) of the dielectric constant for 3D SnSe doped structures (left side) and 2D SnSe doped structures (right side). Figure S4: The measured imaginary part ε_2_ (ω) of the dielectric constant for 3D SnSe doped structures (left side) and 2D SnSe doped structures (right side).
