# Supplementary material for: A Comparative Study of Electronic, Optical, and Thermoelectric Properties of Zn-Doped Bulk and Monolayer SnSe Using Ab Initio Calculations
Source: Nanomaterials (Basel). 2023 Jul 16;13(14):2084. doi: 10.3390/nano13142084 (PMC10383460; doi:10.3390/nano13142084)
Supplement: Supplementary file 1 [file nanomaterials-13-02084-s001.zip › nanomaterials-2440855-supplementary.pdf]

Supplementary Materials

A Comparative Study of Electronic, Optical, and Thermoelectric Properties of Zn-Doped Bulk and Monolayer SnSe Using Ab Initio Calculations

Najwa Al Bouzieh [1], Muhammad Atif Sattar [1,2], Maamar Benkraouda [1], and Noureddine Amrane [1,*]

1 Physics Department, College of Science, United Arab Emirates University (UAEU), Al Ain 15551, United Arab Emirates
2 National Water and Energy Center (NWEC), United Arab Emirates University (UAEU), Al Ain 15551, United Arab Emirates
* Correspondence: namrane@uaeu.ac.ae

3.1 Structural Properties

Table S1. DFT-calculated bond angles of bulk α-SnSe compounds.

Structure	α	β	γ
$\alpha - SnSe$	90.02	89.88	89.97
$Zn_{0.06}\ Sn_{0.94}Se$	90.00	90.10	90.00
$Zn_{0.13}\ Sn_{0.87}Se$	90.03	90.24	90.22
$Zn_{0.19}\ Sn_{0.81}Se$	90.00	90.26	90.00
$Zn_{0.25}\ Sn_{0.75}Se$	90.64	90.92	90.00

Table S2. DFT-calculated bond angles of monolayer α-SnSe compounds.

Structure	α	β	γ
$\alpha - SnSe$	90.00	90.00	90.00
$Zn_{0.06}\ Sn_{0.94}Se$	89.34	90.00	90.00
$Zn_{0.11}\ Sn_{0.89}Se$	88.74	90.00	90.00
$Zn_{0.17}\ Sn_{0.83}Se$	88.17	90.13	90.11
$Zn_{0.22}\ Sn_{0.78}Se$	87.59	90.00	90.00

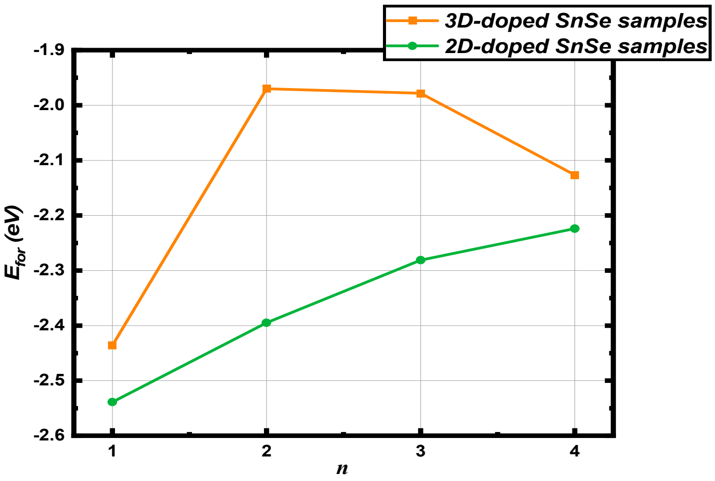

Figure S1: Variation of the formation energy of doped SnSe structures with respect to the number of Zn dopant atoms.

3.2 Electronic Properties

Table S3: Effective Masses (m*) of Charge Carriers at Valence Band Maximum (VBM) and the Conduction Band Minimum (CBM) for the 2D SnSe systems.

	VBM/m_h	CBM/m_e
$\alpha - SnSe$	0.193 (Γ)<br>0.155 (X)	0.158 (X)<br>0.152 (Γ)
$Zn_{0.06}\ Sn_{0.94}Se$	0.639 (Γ-X)	0.220 (X)<br>0.188 (Γ)
$Zn_{0.11}\ Sn_{0.89}Se$	1.033 (Γ-X)	2.417 (X)<br>0.418 (Γ)
$Zn_{0.17}\ Sn_{0.83}Se$	0.889 (Γ-X)	0.499 (Γ)
$Zn_{0.22}\ Sn_{0.78}Se$	0.621 (Γ-X)	0.224 (Γ)

Table S4: Effective Masses (m*) of Charge Carriers at Valence Band Maximum (VBM) and the Conduction Band Minimum (CBM) for the 3D SnSe systems.

	VBM/m_e (holes)	CBM/m_e electrons
$\alpha - SnSe$	1.085 (Z)<br>0.180 (Γ)	0.131 (Γ)<br>0.152 (Y)
$Zn_{0.06}\ Sn_{0.94}Se$	0.234 (Z)<br>0.337 (Γ)	0.218 (Γ)<br>0.236 (Y)
$Zn_{0.13}\ Sn_{0.87}Se$	0.258 (Z)<br>0.243 (Γ)	0.293 (Γ)<br>0.291 (Y)
$Zn_{0.19}\ Sn_{0.81}Se$	0.812 (Z)<br>0.944 (Γ)	0.453 (Γ)<br>0.257 (Y)
$Zn_{0.25}\ Sn_{0.75}Se$	0.735 (Z)<br>0.759 (Γ)	0.420 (Γ)<br>0.261 (Y)

3.3 Optical properties

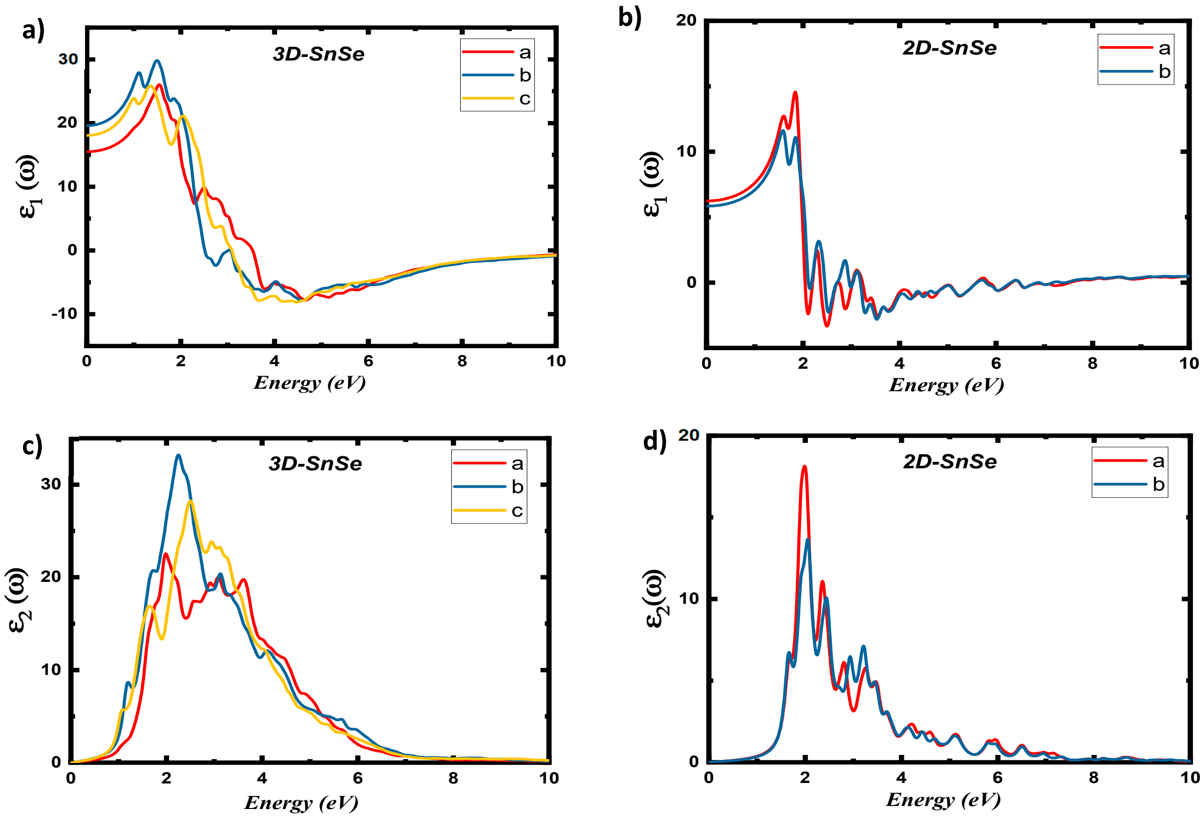

Figure S2: The measured real part $\varepsilon_1(\omega)$ of the dielectric constant (a and b) and the imaginary part $\varepsilon_2(\omega)$ of the dielectric constant (c and d) for 3D SnSe (left side) and 2D SnSe (right side).

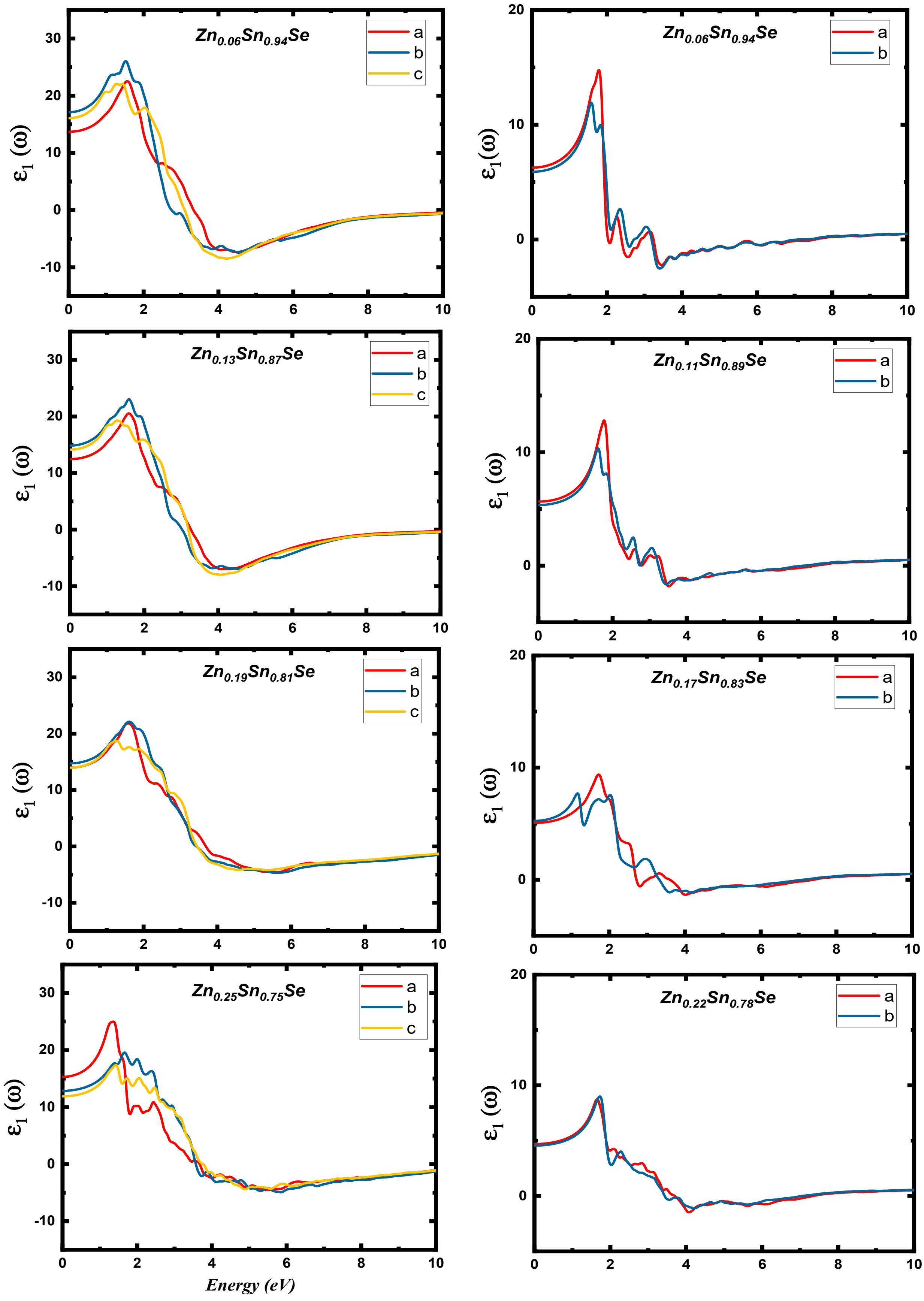

Figure S3: The measured real part $\varepsilon_1(\omega)$ of the dielectric constant for 3D SnSe doped structures (left side) and 2D SnSe doped structures (right side).

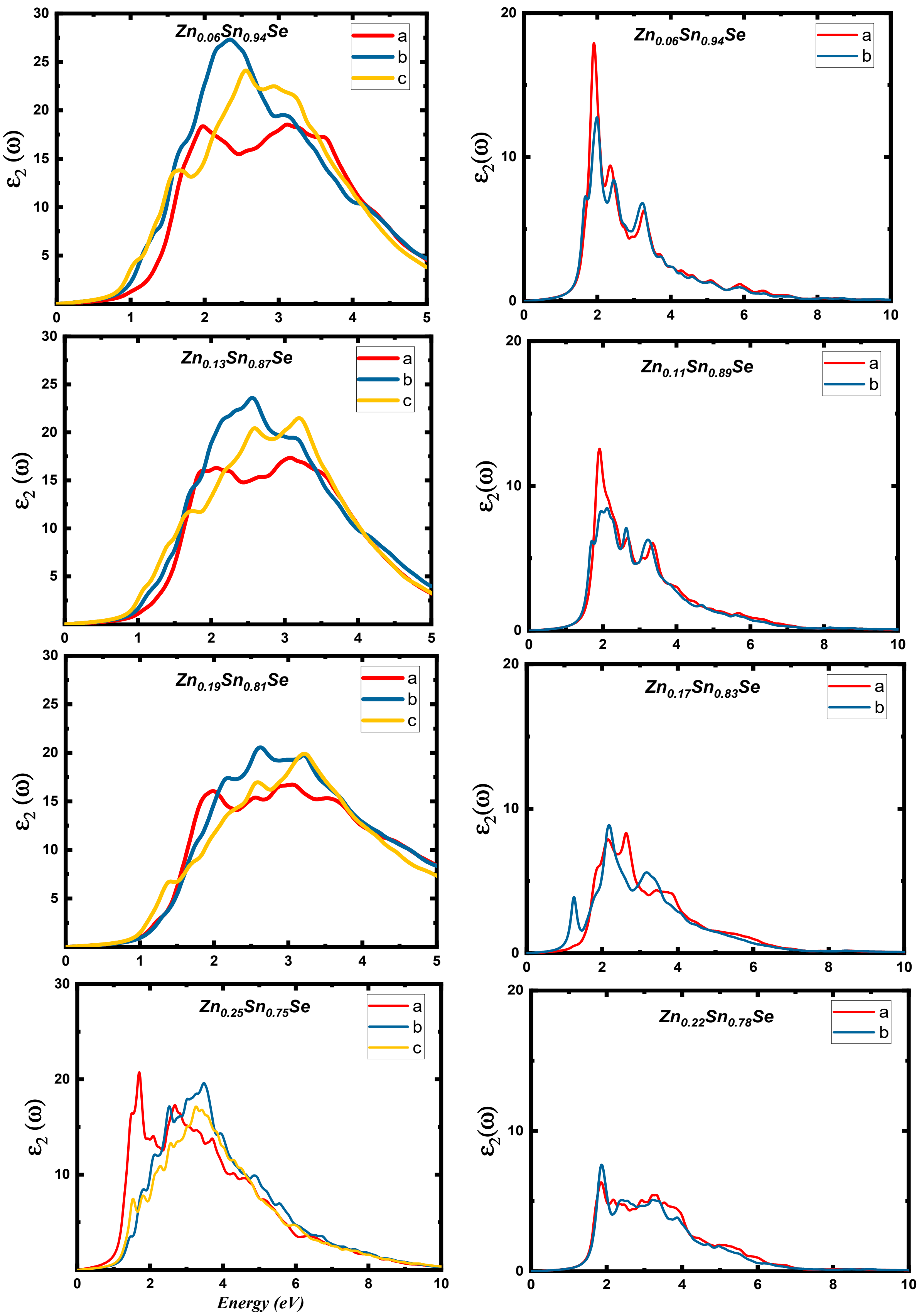

Figure S4: The measured imaginary part $\varepsilon_2(\omega)$ of the dielectric constant for 3D SnSe doped structures (left side) and 2D SnSe doped structures (right side).
